# The effect of encapsulated glutamine on gut peptide secretion in human volunteers

**DOI:** 10.1016/j.peptides.2015.10.008

**Published:** 2016-03

**Authors:** Claire L. Meek, Hannah B. Lewis, Bensi Vergese, Adrian Park, Frank Reimann, Fiona Gribble

**Affiliations:** a The Wellcome Trust-MRC Institute of Metabolic Science, Metabolic Research Laboratories, University of Cambridge, Addenbrooke’s Hospital, Box 289, Hills Road, Cambridge CB2 0QQ, United Kingdom; b Department of Clinical Biochemistry, Cambridge University Hospitals, Addenbrooke's Hospital, Box 281, Hills Road, Cambridge CB2 0QQ, United Kingdom

**Keywords:** AUC, area under the curve, BMI, body mass index, CaSR, calcium sensing receptor, CV, coefficient of variation, DPP-IV, dipeptidyl peptidase-IV, GLP-1, glucagon-like peptide-1, GPCR, G-protein coupled receptor, HV, healthy volunteer, OGTT, oral glucose tolerance test, T2DM, type 2 diabetes mellitus, WHO, World Health Organisation, Glucagon-like peptide 1, Insulin, Nutrients, Incretin, Glutamine

## Abstract

**Context:**

Weight loss and improved blood glucose control after bariatric surgery have been attributed in part to increased ileal nutrient delivery with enhanced release of glucagon-like peptide 1 (GLP-1). Non-surgical strategies to manage obesity are required. The aim of the current study was to assess whether encapsulated glutamine, targeted to the ileum, could increase GLP-1 secretion, improve glucose tolerance or reduce meal size.

**Methods:**

A single-center, randomised, double blind, placebo-controlled, cross-over study was performed in 24 healthy volunteers and 8 patients with type 2 diabetes. Fasting participants received a single dose of encapsulated ileal-release glutamine (3.6 or 6.0 g) or placebo per visit with blood sampling at baseline and for 4 h thereafter. Glucose tolerance and meal size were studied using a 75 g oral glucose tolerance test and ad libitum meal respectively.

**Results:**

In healthy volunteers, ingestion of 6.0 g glutamine was associated with increased GLP-1 concentrations after 90 min compared with placebo (mean 10.6 pg/ml vs 6.9 pg/ml, *p *= 0.004), increased insulin concentrations after 90 min (mean 70.9 vs 48.5, *p *= 0.048), and increased meal size at 120 min (mean 542 g eaten vs 481 g, *p *= 0.008). Ingestion of 6.0 g glutamine was not associated with significant differences in GLP-1, glucose or insulin concentrations after a glucose tolerance test in healthy or type 2 diabetic participants.

**Conclusions:**

Single oral dosing of encapsulated glutamine did not provoke consistent increases in GLP-1 and insulin secretion and was not associated with beneficial metabolic effects in healthy volunteers or patients with type 2 diabetes.

## Highlights

• Weight loss and improved glucose tolerance after bariatric surgery have been attributed to delivery of nutrients to the lower parts of the gut.
• Ileal-release of encapsulated nutrients therefore provides a potential avenue for non-surgical treatment of obesity.
• In a randomised, controlled, blinded clinical study, we assessed the ability of encapsulated ileal-release glutamine to increase concentrations of total glucagon-like peptide 1 (GLP-1), improve glucose tolerance and reduce meal size.
• Encapsulated glutamine did not provide consistent clinically or statistically significant increases in total GLP-1 in healthy volunteers or patients with type 2 diabetes and did not improve glucose tolerance.
• We concluded that higher affinity nutrient agonists may be required to promote GLP-1 secretion.

## 1 Introduction

Over several decades, population levels of obesity, defined as individuals having a body mass index of 30 kg/m^2^ or more, have increased in many countries worldwide. The World Health Organisation now considers obesity to be one of the greatest public health challenges to be faced in the 21st century [1]. Obesity and related conditions, such as type 2 diabetes mellitus (T2DM), are responsible for rising healthcare costs and a great burden of morbidity and mortality. Both obesity and T2DM can be successfully treated with bariatric surgery, although the precise mechanisms responsible for these beneficial effects remain poorly understood. One possibility is that bariatric surgery improves diabetes and aids weight loss by increasing nutrient delivery to the distal gut, which stimulates release of the satiety-promoting incretin hormone glucagon-like peptide 1 (GLP-1). Although GLP-1 mimetics have been given pharmacologically in the treatment of T2DM and obesity [2], an alternative would be to use agents which enhance endogenous GLP-1 production to harness similar benefits with potentially fewer adverse effects.

GLP-1 is produced in enteroendocrine L-cells in the mucosa of the ileum and colon in response to nutrient exposure. Many different proteins or amino acids have been found to stimulate GLP-1 release by interacting with the calcium-sensing receptor (CaSR) [3], [4], GPRC6A [5] or PEPT1 [4]. The amino acid glutamine is abundant in the human diet and a particularly effective stimulant of GLP-1 release in vitro because it not only generates an electrical signal in L-cells but also elevates intracellular cAMP levels [6]. Oral ingestion of glutamine in human volunteers was associated with enhanced GLP-1 secretion and improved glucose tolerance, but a dose of at least 15 g was required [7], [8], possibly due to the low stability of glutamine in gastric acid [9] and its absorption in the upper GI tract resulting in relatively low levels reaching the ileal L-cells. Encapsulation could circumvent this problem by facilitating targeted release of nutrients into parts of the intestine where the L-cells are most abundant.

The aim of the current study was to assess the effect of encapsulated glutamine on GLP-1 concentrations, glucose tolerance and meal size in healthy human volunteers and in individuals with T2DM.

## 2 Methods

### 2.1 Participant recruitment

Healthy male and female volunteers (18–65 years old; body mass index (BMI) 18–35 kg/m^2^) were recruited using advertisements in Addenbrooke’s Hospital and the University of Cambridge. Patients with T2DM (18–65 years old; BMI of 18–40 kg/m^2^) were identified through primary care, outpatient clinics and using advertisements. Participants with anemia or other significant active diseases and those who were pregnant or breastfeeding were excluded. Patients with T2DM who had taken insulin or injectable GLP-1 agonists were excluded from the study. Patients who were taking metformin, sulphonylureas or dipeptidyl peptidase-IV (DPP-IV) inhibitors were asked to withhold these for 12, 24 and 72 h prior to the test respectively.

Participants were provided with written information about the study and gave written informed consent prior to participation. The study was given ethical approval by the Norfolk & Norwich Research Ethics Committee (Reference 12/EE/0389, 25/09/2012; ISRCTN 10757078).

Participants attended the clinical research facility in the morning following an overnight fast. The evening before each visit, participants had a standardized pasta meal (15% protein, 30% fat, 55% carbohydrate) designed to provide 33% of their daily calorie requirement based upon an estimation of their metabolic rate and activity levels [10].

### 2.2 Capsule production

Capsule development and GMP manufacture was performed by Encap Drug Delivery Ltd. (Livingston, UK). Each capsule contained 600 mg of glutamine or 300 mg microcrystalline cellulose (placebo). The capsules were manufactured with an enteric coating designed to promote capsule release 20 min after exposure to an alkaline environment.

### 2.3 Study design

The capsules were tested in a series of blinded, controlled studies in human volunteers. The primary endpoint was the effect of the capsules upon circulating concentrations of total GLP-1 in venous blood. The secondary endpoints were safety, the effect of the capsules upon glucose tolerance, meal size and subjective feelings of hunger and fullness.

#### 2.3.1 Fasting study

Initially, a single-blinded, non-randomised, placebo-controlled dose-ranging study in four healthy volunteers was performed to ascertain the optimal dose for further evaluation and provide preliminary safety data. The doses studied included placebo (10 capsules) and 0.6 g, 1.8 g. 3.6 g and 6.0 g glutamine supplemented where necessary with placebo capsules to preserve participant blinding. The remainder of the study used the two most promising doses, 3.6 and 6.0 g Glutamine in comparison to placebo in a double-blind, controlled randomised design.

On each visit, venous blood was taken at baseline and at intervals following capsule ingestion for evaluation of GLP-1 concentrations. Participants recorded adverse events in a symptom diary and completed questionnaires evaluating hunger, fullness and nausea. On one visit, participants had a DXA scan. Ten healthy volunteers were recruited for evaluation of the primary endpoint in the fasting comparison between placebo, 3.6 g Glutamine and 6.0 g Glutamine.

#### 2.3.2 Glucose tolerance and meal size in healthy volunteers and patients with T2DM

For evaluation of the effect on the capsules on glucose tolerance, nine healthy volunteers and eight patients with T2DM were recruited. The study visits were similar to the fasting study except that each participant had a 75 g oral glucose tolerance test (OGTT) at 90 min after capsule ingestion.

#### 2.3.3 Meal size in healthy volunteers

For evaluation of the effect of the capsules on meal size, ten healthy volunteers were recruited to attend the Clinical Research Facility on three occasions for an ad libitum breakfast given 120 min after capsule ingestion. The ad libitum meal consisted of muesli with chopped fruit, nuts and milk in a homogenous mixture designed to provide 15% protein, 30% fat and 55% carbohydrate. A portion of 1.5 kg (2400 kcal) was provided on each occasion and participants were advised to eat until they felt full. The total amount of food eaten was measured using a universal eating monitor.

### 2.4 Questionnaire design

Hunger, fullness and nausea were assessed using a visual analog scale which has been widely used in the medical literature [11].

### 2.5 Analytical methodology

For analysis of total GLP-1, samples were taken into EDTA plasma tubes, placed on ice immediately after venesection and centrifuged (3500 × *g* at 4 °C 10 min), aliquotted and frozen using dry ice within 15 min of venesection. Samples were stored at −80 °C prior to batch analysis in duplicate of GLP-1 using the Mesoscale Discovery Total GLP-1 kit, a sandwich immunoassay with electrochemiluminescence detection which measures all endogenous forms of GLP-1. This method has a range of 1.4–1000 pg/ml and coefficients of variation (CVs) of 5–7%.

For biochemistry testing, serum samples were allowed to clot for 10 min then centrifuged, separated and frozen within 30 min of venesection and stored at −80 °C prior to analysis. Glucose, alanine aminotransferase (ALT) and creatinine were measured in a clinically-accredited laboratory at Addenbrooke’s hospital using a Siemens’ Dimension analyzer with CVs of <2% within the reference range. Thyroid stimulating hormone (TSH) was measured using a Bayer ADVIA Centaur immunoassay system with CVs of <6% within the reference range. HbA1c was measured on fresh whole blood using a Tosoh analyser with CVs of <5% within the reference range.

For measurement of insulin, samples were collected into lithium heparin plasma tubes, placed on ice, centrifuged (3500 *× g* at 4 °C 10 min), aliquotted and frozen using dry ice within 15 min of venesection. Samples were stored at −80 °C prior to batch analysis using the Diasorin Liaison, an immunoassay with chemiluminescent detection. This method has a range of 20–3470 pmol/l and intra-assay CVs of <5%.

### 2.6 Statistical analysis

Based on previous work [7], using a paired study design, it was estimated that observing 8 subjects per comparison would have 90% power to detect an average difference of 6.0 pmol/l (19.8 pg/ml) of GLP-1. For each endpoint, 8–10 participants were recruited to allow for participant withdrawal. Plasma concentrations of hormones and analytes in the text and figures are shown as mean ± SEM.

As this was a cross-over study, each participant was given placebo and active regimens in randomised order, using a pre-prepared protocol developed using a random number generation package. Paired *t* tests were used to compare plasma levels of GLP-1, glucose and insulin and reported levels of hunger, fullness and nausea according to a visual analog scale. Peak GLP-1 was defined as the maximum concentration of GLP-1 for an individual on a single study day. Peak GLP-1 was defined as the maximum concentration of GLP-1 for an individual on a single study day. Linear regression was used to assess the differences between GLP-1, glucose and insulin concentrations between groups. STATA/SE 13.1 was used for analysis.

## 3 Results

32 participants were recruited (24 healthy volunteers and 8 patients with T2DM). The baseline characteristics of the participants are listed in Table 1. Most healthy volunteers were aged 22–30 years old with a body mass index of 20–25 kg/m^2^ and around 25% body fat. As expected, the patients with T2DM were generally older and had a higher BMI, percentage fat mass and HbA1c compared to healthy volunteers. Most participants had normal hemoglobin, white cell count, ALT, TSH and creatinine at baseline.

### 3.1 Dose ranging study: assessment of the dose-related effects of glutamine capsules on circulating concentrations of total GLP-1 in fasting healthy volunteers

**Fig. 1:**
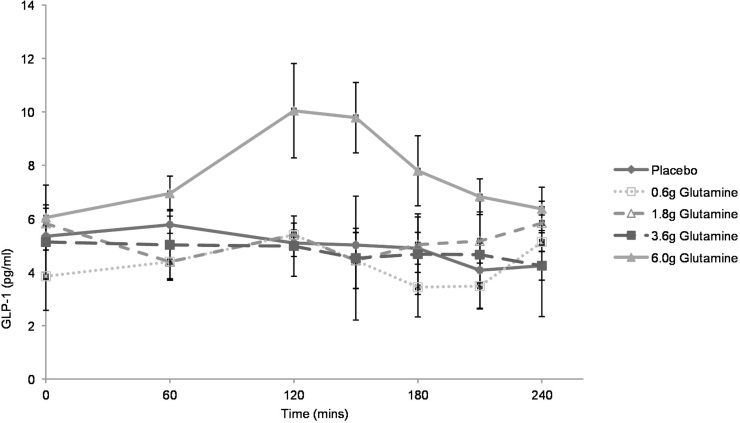
Results of the dose ranging study. 4 blinded healthy volunteers attended on 3–4 occasions for administration of increasing doses of glutamine. The doses chosen for further study were 3.6 g and 6.0 g in comparison to placebo. Capsules were administered at time 0, immediately after baseline blood samples were taken. Data represent mean ± SEM.

Four healthy volunteers were recruited to identify the optimal dose for further study. The highest dose, 6.0 g Glutamine, was associated with a significant increase in peak GLP-1 concentration compared to placebo during (Fig. 1). Based on these results, the doses chosen for further study were 3.6 g Glutamine and 6.0 g Glutamine in comparison to placebo.

### 3.2 Assessment of the effect of glutamine capsules on circulating concentrations of total GLP-1 in healthy volunteers

**Fig. 2:**
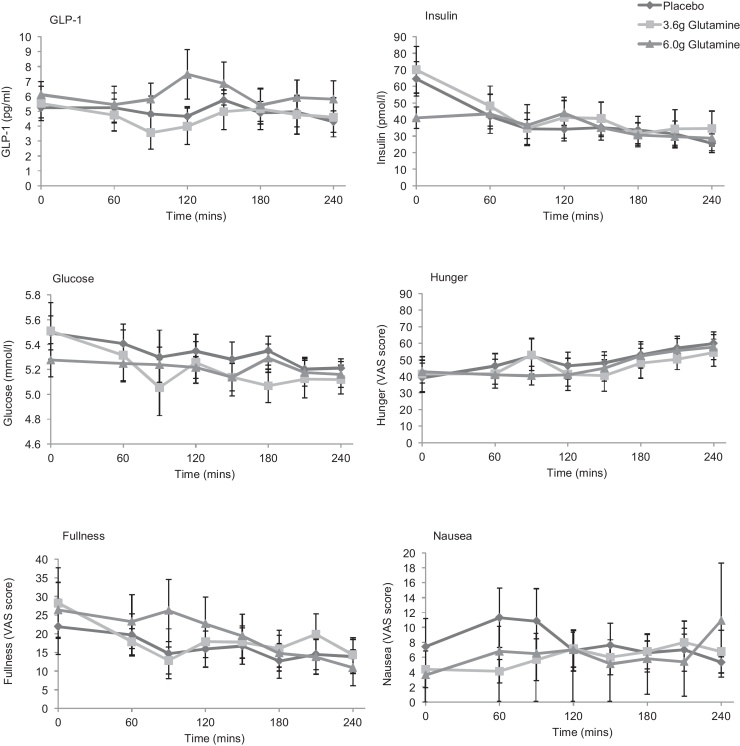
Effect of encapsulated glutamine upon concentrations of total GLP-1, insulin and glucose, and subjective measurements of hunger, fullness and nausea in fasting subjects. Capsules were administered at time 0, immediately after baseline blood samples were taken. 10 participants attended on 3 occasions and took either placebo, 3.6 g Glutamine or 6.0 g Glutamine in a randomised, blinded, cross-over design. Data represent mean + SEM.

Ten healthy volunteers were recruited to assess the effect of glutamine capsules on GLP-1 concentrations (Table 1; Fig. 2). Ingestion of 6.0 g Glutamine was associated with an increase in peak GLP-1 (mean ± SEM: 7.5 ± 4.1 pg/ml placebo vs 10.3 ± 2.6 pg/ml with 6.0 g Glutamine; *p* < 0.001). The area under the curve (AUC) for GLP-1 (from 0 to 240 min) was not significantly different (1217 ± 154 vs 1484 ± 241 pg × min/ml for placebo and 6.0 g Glutamine respectively). There was no significant difference in peak or AUC glucose, insulin, hunger, fullness or nausea between the regimens.

These results were different from those found during the dose-ranging study (Fig. 1). Further examination of individual participants’ data showed one group (*n *= 7) with increased GLP-1 concentrations (responders) while other participants (*n* = 3) showed no evidence of a GLP-1 response. In responders, the shape of the GLP-1 curve was heterogeneous with marked differences between participants. There was also marked intra-individual variation in fasting GLP-1 concentrations in some participants between each of the three visits which was not explained by altered diet or exercise patterns within 24 h of the study visit.

### 3.3 Assessment of the effect of glutamine capsules on glucose tolerance in healthy volunteers

**Fig. 3:**
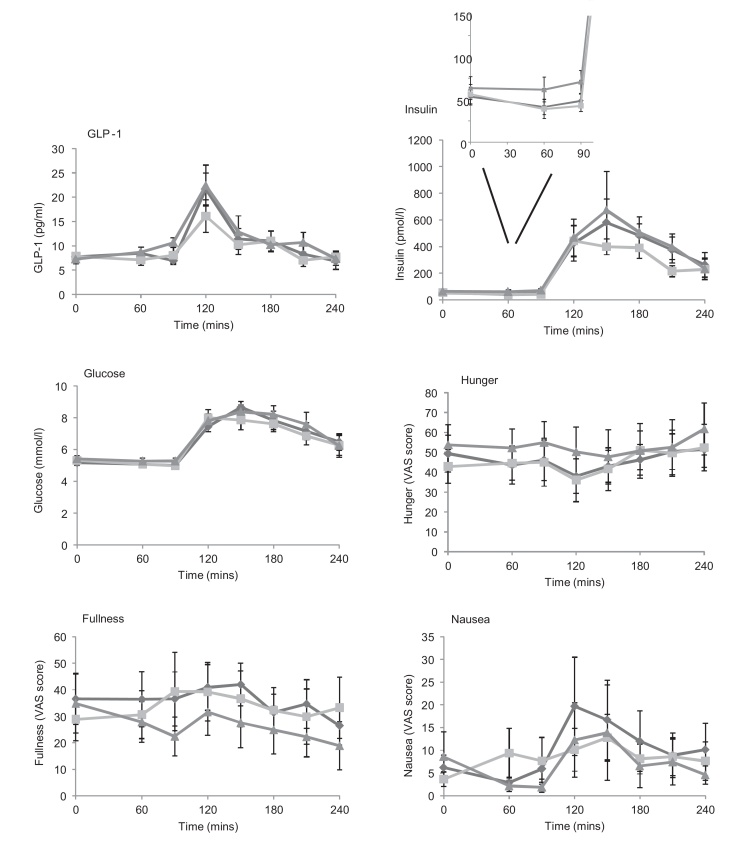
Effect of encapsulated glutamine upon concentrations of total GLP-1, insulin and glucose, and subjective measurements of hunger, fullness and nausea in healthy subjects before and after an oral glucose tolerance test (OGTT) which was given immediately after the 90 min sampling. Capsules were administered at time 0, immediately after baseline blood samples were taken. 10 participants attended on 3 occasions and took placebo, 3.6 g Glutamine or 6.0 g Glutamine in a randomised, blinded, cross-over design. Data represent mean + SEM.

Nine healthy volunteers were recruited to assess the effect of glutamine capsules on glucose tolerance using an oral glucose tolerance test (OGTT; Table 1; Fig. 3). After ingestion of 6.0 g Glutamine, participants had significantly higher concentrations of insulin (mean ± SEM: 70.9 ± 13.4 vs 48.5 ± 7.8 pmol/l; *p* = 0.048) and GLP-1 (10.6 ± 1.0 vs 6.9 ± 0.8 pg/ml; p = 0.004) at 90 min, i.e. immediately prior to ingestion of the 75 g glucose load. No significant difference was seen in the area under the curve before or after the OGTT for glucose, GLP-1, insulin, hunger, fullness or nausea.

### 3.4 The effect of encapsulated nutrients on concentrations of GLP-1, glucose and insulin in patients with type 2 diabetes

**Fig. 4:**
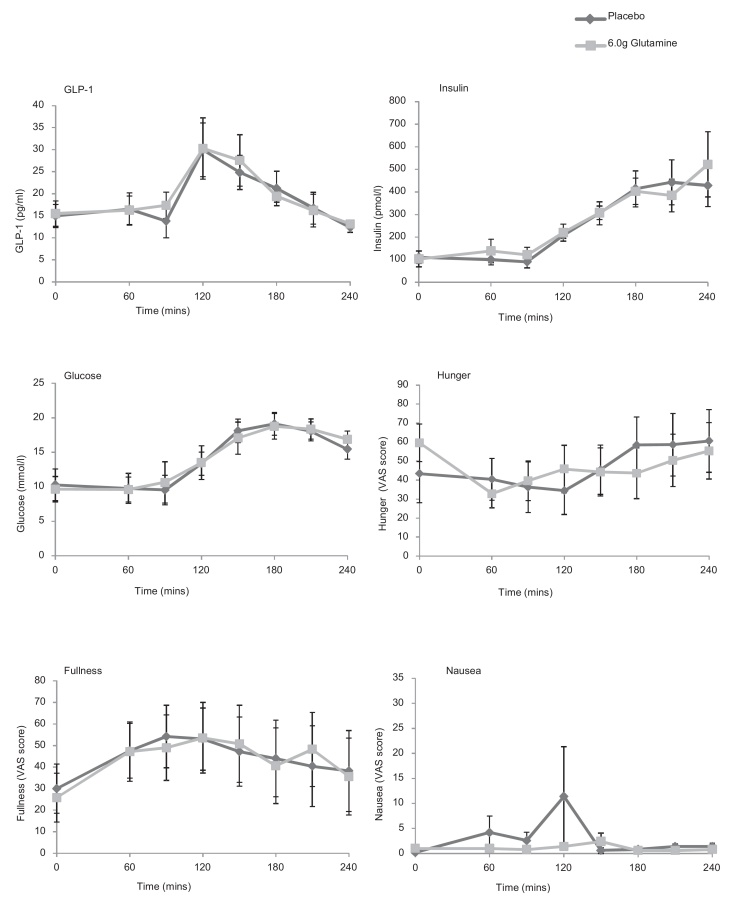
Effect of encapsulated glutamine upon concentrations of total GLP-1, insulin and glucose, and subjective measurements of hunger, fullness and nausea in subjects with type 2 diabetes before and after an oral glucose tolerance test (OGTT) which was given immediately after the 90 min sampling. Capsules were administered at time 0, immediately after baseline blood samples were taken. 8 participants attended on 2 occasions and took either placebo or 6.0 g Glutamine in a randomised, blinded, cross-over design. Data represent mean ± SEM.

Eight patients with T2DM were enrolled into the study and attended for two visits for the assessment of hormone concentrations and glucose tolerance using an OGTT given at 90 min (Table 1; Fig. 4) after placebo or 6.0 g Glutamine. There were no significant differences in peak concentrations of glucose, GLP-1 or insulin or in reported levels of hunger, fullness or nausea. Area under the curve before or after the OGTT for glucose, GLP-1 and insulin were similar between active and placebo treatment regimens.

### 3.5 Assessment of the effect of glutamine capsules on meal size

**Fig. 5:**
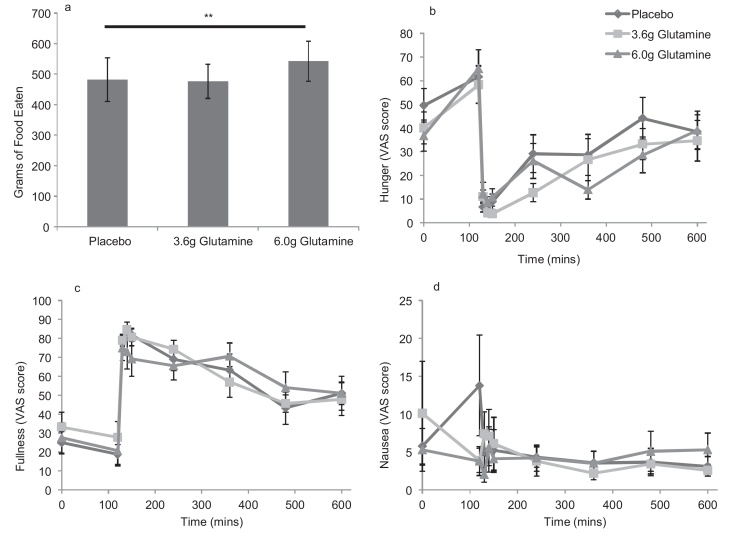
The effect of encapsulated glutamine on meal size, hunger, fullness and nausea in healthy volunteers. Capsules were administered at time 0, immediately after baseline blood samples were taken. An ad libitum meal was consumed 120 mins later. a: Volunteers ate significantly more following ingestion of the 6.0 g Glutamine capsules compared to placebo. b–d: Assessment of hunger, fullness and nausea during and after the ad libitum meal. Data are presented as mean ± SEM. The symbol ** denotes significance at *p* < 0.01 respectively using paired *t* test analysis comparing the 6.0 g Glutamine dose with placebo.

Ten healthy volunteers were recruited to assess the effect of glutamine capsules on meal size (Table 1). The group receiving 6.0 g Glutamine ate significantly more food during the ad libitum meal compared to the placebo group (mean 542 ± 65 g eaten vs 481 ± 71 g, *p* = 0.008; Fig. 5) and had reduced hunger scores postprandially (reduced area under the curve from 120 to 480 min, *p *= 0.053; and 0 to 480 min, *p *= 0.031).

This finding was unexpected and contrary to the hypothesis. Unfortunately, no blood samples were taken from participants during the meal visit. However, participants had significantly higher hunger scores at 120 min following ingestion of 6 g Glutamine, before beginning the meal. On linear regression analysis, hunger at 120 min was linearly related to the amount eaten when the model was adjusted for gender (coefficient 3.41, 95% confidence interval 0.12–6.7, *p *= 0.042).

### 3.6 Assessment of the effect of glutamine capsules on hunger and satiety

Overall, there was no significant difference between peak or AUC for hunger, fullness or nausea for these variables between the three different regimens (Fig. 2, Fig. 3, Fig. 4) except during the meal study (Fig 5). Hunger and fullness were linearly related and hunger scores tended to be lower where nausea scores were high (Fig. S1).

## 4 Discussion

This study demonstrates that up to 6 g of encapsulated glutamine is unable to provoke a physiologically or clinically significant increase in plasma total GLP-1 concentrations in fasting healthy volunteers and is not associated with beneficial effects upon glucose tolerance or meal size. As evident by comparing the results shown in Fig. 1, Fig. 2, some individuals showed small responses to capsule ingestion. However, the magnitude of these responses is likely to be inadequate to cause clinically beneficial effects.

This study used a double-blind, randomised, placebo-controlled, cross-over design to reduce bias and the effects of inter-individual variation. The encapsulated glutamine was tested in healthy individuals and in patients with type 2 diabetes. The capsules were designed to release their contents around 20 min after exposure to an alkaline medium, corresponding broadly to the upper-to-mid ileum. Gastrointestinal transit has not been measured and is likely to vary between individuals, but the capsules were all taken in the fasting state in order to minimize variation in transit time through the stomach. It is possible that not all capsules left the stomach at the same time, which might result in a different profile of glutamine exposure along the gastrointestinal tract and may in part explain inter-individual differences in response. Due to inter and intra-individual variations in gastrointestinal motility, gastric and intestinal secretion volumes and capsule opening profiles, it is not possible to know what concentration of glutamine was reached in the intestinal lumen. These factors might explain why the small GLP-1 responses to capsule ingestion observed in some subjects were not apparent in others. Theoretically, the dissolution of ten capsules simultaneously could trigger a sharp but short-lived GLP-1 response while more gradual dissolution of the capsules may give a more gradual GLP-1 release profile. It is likely, however, that the dose of glutamine was the limiting factor in this experiment. As ten capsules were required to give a dose of 6 g Glutamine, further dose increases were not considered feasible within this study design.

The study was powered according to published data and had 90% power to detect an average difference in GLP-1 of 6.0 pmol/l (20 pg/ml). However, as the difference in peak GLP-1 seen in the fasting state during this study was much lower than this, our study was not powered to detect such small effect sizes. It also remains unclear what levels of GLP-1 might be required to provoke a meaningful change in clinical endpoints such as glucose tolerance and weight reduction. In this study, in the fasting state, GLP-1 concentrations peaked at ∼10 pg/ml, well below the concentrations expected following even a small meal [12]. Indeed, GLP-1 concentrations rose much higher to 20–35 pg/ml after the OGTT. Bariatric surgery has been associated with postprandial total GLP-1 concentrations of 100–160 pmol/l (330–520 pg/ml) [13].

Glutamine is known to be associated with increased GLP-1 secretion and beneficial metabolic effects both in healthy volunteers and in patients with type 2 diabetes. Glutamine is known to elevate cAMP and cytosolic calcium in ileal L-cells and is likely to bind to the calcium sensing receptor (CASR) [14] and the sodium-dependent transporters such as SLC38A2 and SLC6A19 [6]. L-cells also express a range of alternative G-protein coupled receptors, which might be targetable by dietary nutrients or high affinity ligands [15]. As the EC50 for glutamine-triggered GLP-1 secretion in vitro was 0.2 mM [6], we speculate that the dose of glutamine delivered in the capsules was too low to activate L-cells along a large length of the small intestine. Previous work by Greenfield and colleagues has shown that 30 g of oral glutamine in 300 ml water can stimulate increases in GLP-1 and insulin in healthy and obese volunteers and patients with type 2 diabetes [7] with mean peak GLP-1 concentrations of around 22 pmol/l (72 pg/ml) in lean healthy volunteers. This has led to the hypotheses that glutamine could be developed therapeutically in patients with impaired glucose tolerance or diabetes [7]. However, the mode of delivery in that study was challenging as 30 g of glutamine is incompletely soluble in 300 ml water and a ‘swish and swallow’ approach was required [7], [16]. Furthermore, 30 g Glutamine has a calorie content of around 120 kcal and providing additional calories to patients with obesity or type 2 diabetes may exacerbate glucose tolerance through weight gain. The aim of the current approach was to identify if increases in gut hormone concentrations and beneficial metabolic effects could be harnessed using low doses of glutamine using an alternative delivery method which aimed to maximize the exposure of glutamine to lower intestinal L-cells. This approach was not, however, associated with an increase in circulating GLP-1 concentrations comparable to the magnitude of effects seen in Greenfield’s study. Glucose concentrations were also not affected by glutamine ingestion in this study, a finding which is consistent with Greenfield’s work [7]. Chang and colleagues tested an alternative method of delivery involving intra-duodenal infusion of 7.5–15 g Glutamine [17]. They found relatively small increases in GLP-1 (in healthy volunteers and patients with T2DM) and insulin (T2DM only) although their participants demonstrated higher baseline total GLP-1 concentrations than those seen in this study, possibly due to the different analytical methods used for GLP-1 analysis. Their finding of low GLP-1 responses to the 7.5 g Glutamine infusion is consistent with our results, and together both reports suggest that the dose of glutamine is limiting, even when administered directly into the small intestine.

One unexpected finding in our study was the increase in meal size which occurred following ingestion of glutamine in comparison to placebo. As GLP-1 is generally considered to be anorexigenic, and 6.0 g glutamine has a caloric value of around 30 kcal, an association between glutamine and reduced meal size might have been expected rather than the converse. Although blood tests were not taken during the meal test, participants who received the 6.0 g glutamine dose prior to an OGTT had slightly higher circulating concentrations of GLP-1 and insulin prior to the start of the OGTT compared to individuals who received placebo (Fig. 3). As insulin is considered to be orexigenic [18], it is possible that the increased insulin may explain the increased meal size consumed by participants. It is possible, however, that other gut hormones or targets of glutamine might play a role.

No significant difference between concentrations of GLP-1, insulin or glucose after consumption of the glutamine capsules in comparison to placebo were found in subjects with type 2 diabetes. In patients with diabetes, responses may be complicated by altered background GLP-1 secretory patterns [19], [20], gastric emptying [21], or intestinal responsiveness to amino acids in patients taking metformin [22].

In conclusion, orally-administered glutamine capsules did not provoke a consistent increase in GLP-1 or insulin in human volunteers and was not associated with beneficial effects upon glucose tolerance or meal size. We speculate that the low dose of glutamine that could be administered in capsular form was limiting in this study, and suggest that the success of future capsule-based approaches to trigger GLP-1 release will be dependent, at least in part, on the choice of higher affinity ligands to receptors localised on L-cells. Further work is needed to identify if other nutritional products could function as more potent agonists to promote endogenous GLP-1 secretion and harness its clinical benefits.

## Funding

This project was supported by a project grant from the European Union Seventh Framework Programme (FP7/2007–2013; grant agreement no. 266408) as part of a larger collaboration called Full4Health. Claire Meek receives salary funding from the Wellcome Trust Translational Medicine and Therapeutics Programme which is funded by the Wellcome Trust in association with Glaxo SmithKline. FMG and FR were funded by the Wellcome Trust (WT088357/Z/09/Z and WT084210/Z/07/Z.)

## Duality of interest

Claire Meek receives salary funding from the European Union Seventh Framework Programme (FP7; grant agreement no. 266408) and from the Wellcome Trust Translational Medicine and Therapeutics Programme which is funded by the Wellcome Trust in association with Glaxo SmithKline. FMG is a member of the external scientific advisory board for BioKier, who develop encapsulated nutrients for the treatment of metabolic diseases.

CLM is the guarantor of this work and, as such, had full access to all the data in the study and takes responsibility for the integrity of the data and the accuracy of the data analysis.

## Contribution statement

CLM designed the study, obtained regulatory approvals for the study, recruited volunteers, supervised capsule development and organised the participant visits. She also coordinated the safety reporting and provided medical cover for participants in case adverse events occurred, performed the data analysis and wrote the final report. HBL assisted with participant recruitment and statistical analysis and reviewed the final manuscript. BV assisted with participant recruitment and reviewed the final manuscript. AP assisted with study design, advised on regulatory matters and participant recruitment and reviewed the final manuscript. FR contributed to study design, data analysis and reviewed and revised the final manuscript with contributions to the discussion. FMG identified the study question and designed the study, contributed to the discussion and reviewed and revised the final manuscript.

## Figures and Tables

**Table 1:** Baseline characteristics of study participants—characteristics shown as median (range). HV: healthy volunteers; T2DM: patients with type 2 diabetes mellitus.

	HV: assessment of GLP-1 concentrations *n *= 10	HV: assessment of glucose tolerance *n* = 9	HV: assessment of meal size^a^ *n* = 10	T2DM: assessment of glucose tolerance *n* = 8
Gender	5 male, 5 female	4 male, 5 female	5 male, 5 female	4 male, 4 female
Age (years at enrolment)	31.3 (9.7)	36.2 (10.5)	33.9 (10.2)	57.9 (10.7)
Body Mass Index (kg/m^2^)	22.9 (3.5)	25.0 (3.9)	23.4 (4.1)	32.5 (5.6)
Waist circumference (cm)	79.0 (14.0)	88.3 (11.5)	83.1 (15.5)	104.0 (16.8)
Hip circumference (cm)	94 (10)	102 (8)	96 (11)	120 (10)
DXA percentage fat mass	23.8 (11.3)	29.3 (12.5)	29.1 (12.4)	41.8 (8.0)
Hemoglobin (g/l)	136 (13)	138 (14)	135 (15)	136 (16)
White Cell Count *10^9^/l	5.1 (0.7)	5.3 (1.1)	4.8 (1.0)	6.7 (1.0)
Platelets *10^9^/l	189 (25)	225 (52)	197 (30)	165 (82)
Creatinine (μmol/l)	70.5 (11.8)	67.3 (11.0)	64.0 (8.9)	70.9 (10.4)
ALT (IU/l)	32.5 (23.8)	36.4 (25.9)	33.6 (27.6)	46.0 (23.3)
TSH (U/l)	2.0 (0.8)	2.1 (0.6)	2.0 (0.9)	1.9 (1.0)
HbA1c (mmol/mol)	36.0 (4.2)	36.9 (3.4)	36.4 (3.6)	57.6 (17.6)

a Only 8 of these participants had blood taken and a DXA scan.
