# Supplementary material for: The effect of encapsulated glutamine on gut peptide secretion in human volunteers
Source: Peptides. 2016 Mar;77:38–46. doi: 10.1016/j.peptides.2015.10.008 (PMC4788717; doi:10.1016/j.peptides.2015.10.008)
Supplement: Supplementary file 1 [file mmc1.docx]

Supplementary information: Safety Report

The assessment of safety was a secondary endpoint of the study and was assessed using a symptom diary and visual analogue scale for nausea. There were no serious adverse events during the study although a wide variety of symptoms were reported (table S1).

**Table S1: Symptoms experienced according to treatment regimen given (individual participant identification number given in brackets).**

| Symptoms after Placebo | Symptoms after 6g Glutamine | Symptoms after 3.6g Glutamine |
| --- | --- | --- |
| Dizzy, headache (P2)  Breathlessness & sensation of tachycardia (P2)  Nausea (P13)  Shakiness ?hypoglycaemia (P20)  Nausea and vomiting (P21)  Light-headedness (P23) | Headache (P12)  Headache (P13)  Uncomfortably full & abdominal pain (P14)  Flatulence (P15)  Abdominal discomfort & vomiting (P16)  Felt less hungry for several hours (P20) | Dizzy (P9)  Headache, backache(P12)  Constipation (P12) |

Results from the symptom diaries showed that side-effects were experienced following administration of all regimens (Table S1). The vast majority of symptoms were classified as mild (1-3/10 on a visual analogue scale).

Symptoms tended to fall within 3 broad categories:

- Symptoms related to the protocol, commonly associated with fasting. These included mild headache, dizziness and light-headedness and were broadly similar across all treatment groups.
- Gastrointestinal symptoms occurred including nausea, vomiting, constipation, flatulence and abdominal discomfort. Most symptoms within this category were mild. Two participants experienced vomiting episodes after placebo and 6g glutamine doses. Two participants specifically mentioned nausea on their symptom diary following ingestion of placebo capsules. One of these participants reported nausea after ingestion of the OGTT and the visit had to be abandoned after emesis of the intact capsules.
- Other events:
  - One participant experienced tremor after the test and attributed her symptoms to hypoglycaemia, although no testing facilities were available as she had left the hospital. The symptoms resolved quickly after eating and occurred after a placebo visit.
  - One participant experienced breathlessness and the sensation of tachycardia after her first visit. On assessment of her observations including saturations, clinical examination and electrocardiogram, no concerning abnormalities were identified. These symptoms occurred during a placebo visit and resolved quickly after reassurance.

When using the visual analogue scores, participants put a cross on a 100mm line to indicate how hungry, full or nauseated they felt at each timepoint. As expected, there was a small negative association between nausea and hunger, and a strong negative correlation between hunger and fullness (fig. S1). There was a small significant association between GLP-1 concentrations and nausea (fig. S1). Most participants experienced no nausea during the study.

Figure S1: Relationships between visual analogue scores and/or GLP-1 concentrations in all participants at all timepoints excluding the meal visits when blood was not was taken. A: Most participants experienced no nausea during the test. There was a small but significant positive relationship between GLP-1 concentration and nausea scores. B: There was a small negative correlation between nausea and hunger. C: There was a strong negative correlation between hunger and fullness. Statistical analysis was performed using linear regression.

In conclusion, side effects occurred in both glutamine and placebo groups. Fasting was associated with dizziness, headache and light-headedness. Gastrointestinal symptoms were common in active and placebo groups with no particular evidence of clustering in the 6.0g glutamine group. However, there was a small but significant relationship between nausea and total GLP-1 concentrations.
